# Effects of biochar and organic-inorganic fertilizer on pomelo orchard soil properties, enzymes activities, and microbial community structure

**DOI:** 10.3389/fmicb.2022.980241

**Published:** 2022-08-03

**Authors:** Yang Song, Quan Zhao, Xiuzhu Guo, Izhar Ali, Fayong Li, Shaosheng Lin, Dongfeng Liu

**Affiliations:** ^1^Institute of Subtropical Crops of Zhejiang Province, Wenzhou, China; ^2^College of Agriculture, Guangxi University, Nanning, China

**Keywords:** biochar, organic-inorganic fertilizer, soil enzymes activities, bacteria, pomelo orchard

## Abstract

Fertilizer management can influence soil microbes, soil properties, enzymatic activities, abundance and community structure. However, information on the effects of biochar in combination with organic-inorganic fertilizer after 3 years under pomelo orchard on soil bacterial abundance, soil properties and enzyme activities are not clear. Therefore, we conducted a field experiment with seven treatments, i.e., (1) Ck (control), (2) T1 (2 kg biochar plant^–1^), (3) T2 (4 kg biochar plant^–1^), (4) T3 (2 kg organic-inorganic mixed fertilizer plant^–1^), (5) T4 (4 kg biochar + 1.7 kg organic-inorganic mixed fertilizer plant^–1^), (6) T5 (4 kg biochar + 1.4 kg organic-inorganic mixed fertilizer plant^–1^), and (7) T6 (4 kg biochar + 1.1 kg organic-inorganic mixed fertilizer plant^–1^). The soil microbial communities were characterized using high-throughput sequencing of 16S and internal transcribed spacer (ITS) ribosomal RNA gene amplicons. The results showed that biochar combined with organic-organic fertilizer significantly improved soil properties (pH, alkali hydrolysable nitrogen, available phosphorus, available potassium, and available magnesium) and soil enzymatic activities [urease, dehydrogenase (DHO), invertase and nitrate reductase (NR) activities]. Furthermore, soil bacterial relative abundance was higher in biochar and organic-inorganic treatments as compared to control plots and the most abundant phyla were Acidobacteria (40%), Proteobacteria (21%), Chloroflexi (17%), Planctomycetes (8%), Bacteroidetes (4%), Verrucomicrobia (2%), and Gemmatimonadetes (1%) among others. Among the treatments, *Acidothermus, Acidibacter*, *Candidatus Solibacter* and *F473* bacterial genera were highest in combined biochar and organic-inorganic treatments. The lowest bacterial abundance and bacterial compositions were recorded in control plots. The correlation analysis showed that soil attributes, including soil enzymes, were positively correlated with Chloroflexi, Planctomycetes, verrucomicrobia, GAL15 and WPS-2 bacterial abundance. This study demonstrated that biochar with organic-inorganic fertilizer improves soil nutrients, enzymatic activities and bacterial abundance.

## Introduction

Pomelo is a vital citrus fruit, and its farming has been steadily increasing worldwide in recent years (Chen et al., 2020). China is the world’s top pomelo producer, accounting for 52% of worldwide pomelo production in 2017 (Gennari et al., 2019). In China’s Zhejiang Province, pomelo species are mainly cultivated in western and southeastern Zhejiang mountainous areas. The soil is basically considered acidic with poor soil fertility (Chen et al., 2022). To improve pomelo fruits yield, farmers use excessive use of chemical fertilizers, including nitrogen, phosphorus, and potassium (Yan et al., 2022), which results in soil acidification, destroying soil structure, reducing soil organic matter, and inhibiting the absorption of soil nutrients (Iqbal et al., 2021; Ali et al., 2022). Soil nutrient status is the most critical factor which affects soil microbes (Paul, 2014).

Soil microbes play a significant role in soil quality and agricultural production in the soil ecosystem (Khan A. et al., 2021, 2022). These bacteria perform soil important functions, including nutrient recycling, organic matter breakdown, soil structure building, secretion of plant growth stimulants, degradation of organic pollutants, and insect and disease suppression (Kirchman, 2018; Khan I. et al., 2022; Muhammad et al., 2022). Furthermore, fertilizer management directly altered soil microbial abundance and community composition structure (Li et al., 2012). While the excessive use of chemical fertilizer cause soil acidification, which reduces the most beneficial microorganisms suitable for neutral environments, which is not conducive to the transformation of soil nutrients (Ji et al., 2020). Moreover, to reduce the soil’s acidity, orchard farmers apply a large amount of quicklime, which further leads to soil compaction, and adequate nutrients such as magnesium, calcium, and zinc in the soil cannot be absorbed by the tree (Morales et al., 2018). A series of problems such as severe loss due to absorption and utilization and soil pollution (Hossain et al., 2021; Huang et al., 2021) has seriously restricted the development of the pomelo industry in Zhejiang province. Alternatively, biochar amendment is well known to improve soil physicochemical properties and plant growth and development under lower chemical fertilization (Ali et al., 2021).

Biochar is a carbon-rich product prepared by pyrolysis of biomass such as straw, sawdust, livestock and poultry manure, and edible fungus residues under anaerobic or low-oxygen conditions (Alburquerque et al., 2013; Ali et al., 2020a; Ullah et al., 2021). Studies have shown that the surface of biochar is rich in oxygen-containing functional groups such as −COOH and −OH, which are primarily alkaline and have a positive effect on acid pH, DOC and bacterial community diversity and soil respiratory enzyme activity (Wang et al., 2014; Chen et al., 2015; Demisie and Zhang, 2015; Xu et al., 2019; Tan et al., 2020; Ihsanullah et al., 2022). Likewise, Wang et al. (2014) found that biochar addition significantly increased soil organic matter and pH, and decreased soil exchangeable acid content in acidified tea gardens. In addition, due to rich carbon content, biochar application into the soil will cause the proliferation of microorganisms and improve the activity of soil enzyme activity (Ullah et al., 2020). Furthermore, the rich pore structure also provides habitats for beneficial soil microorganisms (Palansooriya et al., 2019). Soil condition plays a crucial role in determining microbial community composition in biochar-amended soils (Palansooriya et al., 2019). However, the various rates of biochar in combination with different fertilizers and its application time have different effects on soil pH and microbial communities (Dai et al., 2021). For example, Chen et al. (2013) found that after 1 year of biochar application, bacterial gene copy numbers in mildly acidified paddy soil increased by 28–64%. Furthermore, Ali et al. (2022) reported that biochar application combined with inorganic fertilizer improved soil bacterial abundance and reduced fungi communities. However, some studies have also found that adding biochar reduces soil microbial biomass and the richness of microbial community structure (Dempster et al., 2012).

Therefore, the effects of biochar application combined with organic-inorganic fertilizer under pomelo orchard after 3 years on soil bacterial abundance and community composition are scanty. This study aimed to evaluate the impacts of the combined application of biochar and organic-inorganic fertilizer on soil phytochemical properties, biochemical properties, soil bacterial abundance, and community structure under pomelo acidic soil.

## Materials and methods

### Experimental location

A field experiment was conducted in Bilian Town (28° 19’ N, 120° 33’ E), Yongjia County, Wenzhou City, Zhejiang Province, China. The experimental place’s climate is classified as a subtropical monsoon climate, in which the annual average temperature is 18.3^°^C and the average annual precipitation is 1718.3 mm. The soil type of the experimental field was sandy loam and the primary soil properties of the experimental fields, i.e., pH, organic matter, available nitrogen, available phosphorus, available potassium and available magnesium, were 4.41, 15.4 g kg^–1^, 113.3 mg kg^–1^, 246.1 mg kg^–1^, 370.8 mg kg^–1^, and 30.2 mg kg^–1^, respectively. The tested pomelo trees were 20 years old, and the trees with more consistent growth with the same size were selected to carry out the test.

### Biochar and organic-inorganic fertilizer production

The test biochar is produced from rice straw in a traditional kiln, where the temperature was between 500–600^°^C. The pH, TN, TP, TK and OC of rice straw biochar was 8.68, 17.38 g kg^–1^, 3.67 g kg^–1^, 7.27 g kg^–1^, and 389.56 g kg^–1^. The organic-inorganic compound fertilizer is produced by Zhejiang Zhongci ecological fertilizer Co., Ltd. (organic matter ≥ 25%, N + P_2_O_5_ + K_2_O ≥ 15%).

### Experimental design

The experimental layout was a single-factor randomized block design with 7 treatments. The treatment combinations were as follows (1) Ck (control), (2) T1 (2 kg biochar plant^–1^), (3) T2 (4 kg biochar plant^–1^), (4) T3 (2 kg organic-inorganic mixed fertilizer plant^–1^), (5) T4 (4 kg biochar + 1.7 kg organic-inorganic mixed fertilizer plant^–1^), (6) T5 (4 kg biochar + 1.4 kg organic-inorganic mixed fertilizer plant^–1^), and (7) T6 (4 kg biochar + 1.1 kg organic-inorganic mixed fertilizer plant^–1^). The experiment consisted of four replication and five plants in each replication. Biochar was applied in late February 2018. A 30 cm wide and 20 cm deep annular fertilization ditch was dug near the drip line. Biochar and organic-inorganic compound fertilizer were applied into the ditch and mixed with the soil. The annual fertilizer amount was nitrogen (N) 300 kg hm^–2^, P_2_O_5_ 75 kg hm^–2^, K_2_O 112.5 kg hm^–2^, and organic-inorganic compound fertilizer was used for spring, summer, and autumn fertilization which account for 40, 30, and 30%, respectively of the annual total; biochar was only applied once during the experiment. Conventional orchard management was mainly based on artificial weeding, and disease-pest control with mancozeb, copper sulfate preparations, and abamectin.

### Measurement and analysis

#### Soil properties

The orchard soil used for the experiment was sampled at 0–20 cm depth from each arable site near the drip line, and the plant debris and gravel were removed, and the samples were mixed and placed in refrigeration. Soil pH value was determined by using a digital pH meter (Thunderbolt PHS-3C China) Ali et al. (2022). Soil alkaline hydrolyzable nitrogen (AN) was measured by the alkaline hydrolysis diffusion method (Iqbal et al., 2019). In contrast, available phosphorus (AP) was determined by the ammonium fluoride-hydrochloric acid leaching molybdenum antimony anti-colorimetric method (Yi, 2019). Furthermore, available potassium (AK) was determined by Neutral ammonium acetate extraction flame photometry (Grewal et al., 2017) and ammonium acetate extraction atomic absorption spectrophotometry was used for the determination of available magnesium (AM) (Simard, 1993).

#### Soil enzyme activities measurement

The measurement of soil enzyme activities was followed by the methods of Jin et al. (2009) and Zhang et al. (2011). Soil invertase activity was determined with a sucrose solution as the substrate and incubated for 24 h at 37^°^C. Then the mixture was measured for the produced glucose with a colorimetric method (Gopal et al., 2007). Nitrate reductase activity was measured with 2,4-dinitrophenol solution, potassium nitrate solution and distilled water kept at 25^°^C for 24 h. Then the treated solution was added buffer and color reagent after filtrate and measured by colorimetric method (Singh and Kumar, 2008). Dehydrogenase activity was measured by the reduction reaction of triphenyl tetrazolium chloride to triphenylformazan. Then it was expressed as mg triphenylformazan g^–1^ soil h^–1^ (Xie et al., 2017). The determination of phosphatase activity was used p-nitrophenyl phosphate as substrate and was incubated for 24 h at 37°C. the liberated phenol was determined colorimetrically, and the acid phosphatase activity was expressed as mg phenol kg^–1^ soil h^–1^ (Xie et al., 2017). Urease activity was measured in 5 g of fresh soil using 10% of urea solution and then was kept using 5 ml citrate solution at pH 6.7 and 5 ml substrate for 24 h at 37^°^C. Then filtered 3 ml of 0.9% sodium hypochlorite solution and 4 ml of sodium phenol solution treated with 1 ml filtrate. Then the ammonium was released from urea hydrolysis and it was quantified in an ultraviolet spectrometer subsystem at 578 nm (Iqbal et al., 2022).

#### DNA extraction and sequencing

First of all, the total DNA of soil bacteria was collected with a soil DNA extraction kit (Omega Bio-tek Inc., Doravilla, GA, United States), and the quality of the DNA was checked by 1% agarose gel electrophoresis. Secondly, the bacteria DNA for soil sample was sent to Guangdong Meige Gene Technology Co., Ltd., for high-throughput sequencing. The analysis of soil bacteria high-throughput sequencing data is based on the cloud service of the Illumina MiSeq platform determined by Guangdong Meige Gene Technology Co., Ltd. The project number is MGWH20210317A1NZGQ-1N_A-20210610-9248.

#### Illumina sequencing data processing

Before building a gene segment, the paired reads were spliced to fuse the sequences using FLASH (version 1.2.3) software (Magoc and Salzberg, 2011). Under USEARCH (version 8.1.1861), chimeric sequences were identified and deleted using a *de novo* technique (Edgar, 2010). Following the removal of the chimera, high-quality bacterial genomes were gathered for further investigation. For the statistical analysis that followed, each sample was sub-sampled independently for effective bacterial sequences. The data were then processed using a customized SOP pipeline and the software package QIIME (Quantitative Insights Into Microbial Ecology v1.8.0) under USEARCH (Tian et al., 2015). At 97% sequence identity, the selected sequences were clustered to operational taxonomic units (OTU) using a two-stage clustering technique in USEARCH (version 8.1.1861) (Edgar, 2010). The SILVA reference alignment was used to align representative sequences from each OTU (Yilmaz et al., 2014). RDP assigned taxonomy to each representative sequence with a minimum confidence level of 85 percent.

#### Alpha and beta diversity analysis

An OTU-based analysis method was used to assess bacterial diversity in each sample from each plant (alpha diversity). QIIME software (v1.8.0) was used to calculate OTU richness, Chao1, Simpson, and Shannon indices to measure the diversity index and species richness (alpha diversity) among the genotypes for each sample, with a sequencing depth of 3%. ANOVA with *p-*values was used to examine whether there were significant differences in the diversity indices or species richness among the plant rhizosphere soil samples. At a 97 percent level of OTU similarity, the rarefaction curves and rank abundance curves were generated. The similarity index of community structure among all the samples was determined using beta diversity analysis. Beta diversity was counted at the OTU level of genotypes using weighted UniFrac distances and visualized using principal coordinate analysis (PCoA). QIIME software (v1.8.0) was used to cluster and estimate the weighted UniFrac distance matrices, which revealed phylogenetic relationships among distinct communities as well as their abundance in all samples.

### Statistical analysis

The experimental data analysis was carried out based on the cloud service provided by Guangdong Meige Gene Technology Co., Ltd. (Shenzhen, China). Before data analysis, the data was leveled according to the minimum number of sample sequences. Among them, the diversity index (alpha-diversity and beta diversity) was calculated using Qime software^1^ ; the sample hierarchical clustering and non-metric multi-dimensional analysis were performed using the UPGMA non-weighted group average method. SPSS venison 17 and EXCEL 2010 were used for statistical analysis of test data, and SigmaPlot 12.5 (Systat Software, San Jose, CA, United States) software was used to make charts.

## Results

### Changes in soil properties

Biochar combined with organic-inorganic fertilizer to soil significantly affected soil properties of pomelo orchard (Table 1). Results showed that soil pH was increased by 68% in T6 compared to Ck. AN was higher significantly in T1 and T2 treatments by 11.3 and 6.7%, respectively. In contrast, T4 decreased alkaline hydrolyzable nitrogen by 4.7, 7.7, and 10.4% as compared to T1, T2, and T3, respectively. Furthermore, biochar addition to soil did not affect AP compared to non-biochar treatments, whereas the maximum value was recorded in T3. In addition, an increase of 27.7, 18.9, 42.3, 36.2, 23.2, and 12.3% in AK was recorded in T4, T3, T1, T5, T2, and T6, respectively compared to Ck. In the case of AM, the highest value (80.0 ± 1.32) of AM was recorded in T4 compared to Ck, and no significant difference among T1, T6, T3, and T5 were observed.

**TABLE 1:** Changes of soil pH and mineral element content after application of biochar for 3 years.

Treatment	pH	AN (mg.kg^–1^)	AP (mg.kg^–1^)	AK (mg.kg^–1^)	AM (mg.kg^–1^)
Ck	4.29 ± 0.11d	238 ± 13.0e	477 ± 63.0c	47.0 ± 2.35c	61.0 ± 4.6d
T1	4.98 ± 0.17a	265 ± 6.9c	539 ± 23.8b	60.0 ± 1.32a	70.0 ± 1.32b
T2	5.23 ± 0.34a	254 ± 10.8d	546 ± 75.8b	55.9 ± 0.47b	66.9 ± 5.84c
T3	4.37 ± 0.07d	283 ± 7.4b	595 ± 21.4a	64.9 ± 4.9a	73.6 ± 5.87b
T4	4.58 ± 0.12c	297 ± 17.0a	611 ± 32.8a	66.0 ± 1.32a	80.0 ± 1.32a
T5	4.65 ± 0.14bc	274 ± 8.8bc	556 ± 55.8b	57.9 ± 0.47ab	72.0 ± 1.02b
T6	4.71 ± 0.12b	266 ± 14.9c	568 ± 28.5b	52.8 ± 0.71b	69.8 ± 0.71b

Different letters within the column indicates significant difference at P < 0.05. Ck (control), T1 (2 kg biochar plant^–1^), T2 (4 kg biochar plant^–1^), T3 (2 kg organic-inorganic mixed fertilizer plant^–1^), T4 (4 kg biochar + 1.7 kg organic-inorganic mixed fertilizer plant^–1^), T5 (4 kg biochar + 1.4 kg organic-inorganic mixed fertilizer plant^–1^), T6 (4 kg biochar + 1.1 kg organic-inorganic mixed fertilizer plant^–1^).

### Changes in soil enzymes activities

**FIGURE 1:**
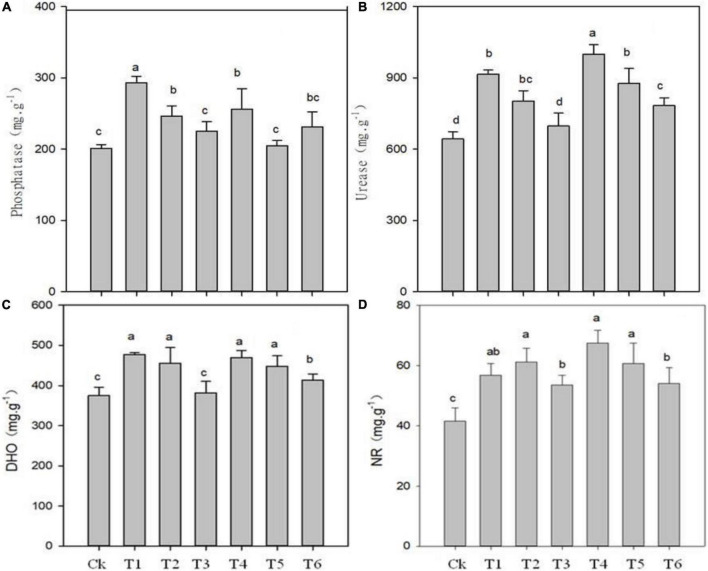
Effects of biochar, organic-inorganic and mixed fertilizer on the soil phosphate **(A)**, urease **(B)**, dehydrogenase (DHO) **(C)** and nitrate reductase (NR) activities **(D)**. Different letters (a, b, and c) above the columns and curve indicate statistical significance at *p* < 0.05.

**FIGURE 2:**
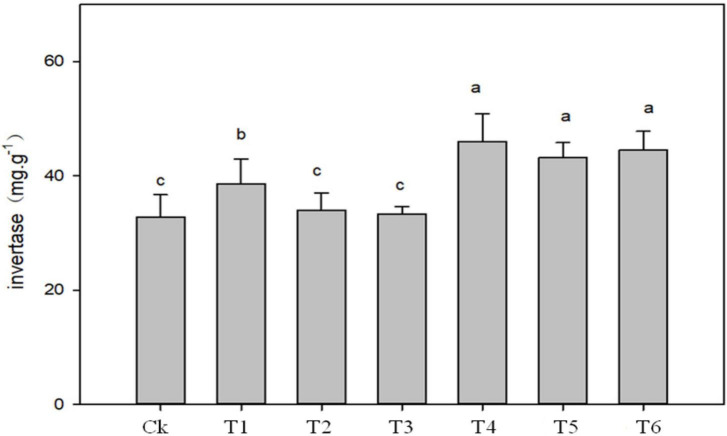
Effects of biochar, organic-inorganic and mixed fertilizer on the soil invertase activity. Different letters (a, b, and c) above the columns and curve indicate statistical significance at *p* < 0.05.

The application of biochar, organic-inorganic and mixed fertilizer significantly affected phosphate, urease, dehydrogenase (DHO) and nitrate reductase (NR) activities in pomelo orchard (Figure 1). Results showed that phosphate activity was significantly affected by sole biochar application and was increased by 45.37 and 14.88% in T1 and T2, respectively, compared to Ck. The treatments T3, T4, T5, and T6 improved phosphatase activity by 9.61, 24.29, 1.19, and 14.03%, respectively, compared to Ck treatment. Urease and invertase activity were significantly higher by 51.39 and 41% in T4, respectively, followed by T1, T5, and T4 over Ck treatment (Figures 1, 2).

Compared to Ck, biochar addition treatments T1, T2, T4, T5, and T6 significantly improved DHO activity by 27, 17, 24.90, 15.54, and 8.81%, respectively (Figure 1C). Similarly, NR activity was increased by 36.60, 34.42, 19.57, 48.10, 28.35, and 20.53% in T1, T2, T4, T5, and T6, respectively (Figure 1D). The lowest values of DHO and NR were observed in Ck (374.74 and 41.59, respectively), followed by T3 treatment. The overall results showed that soil enzymes activities were improved by biochar application over control.

### Sequencing quality control and summary

**FIGURE 3:**
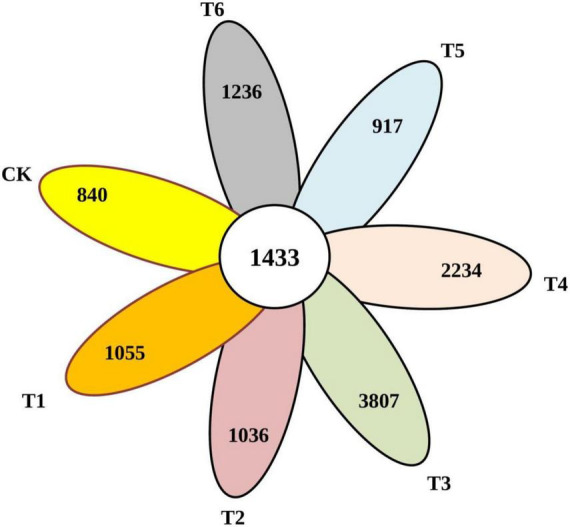
Venn map of common and endemic species in the community.

After the screening, pre-clustering, and chimera removal, a total of 810,347 reads of good quality bacterial 16Sr RNA were recovered, with an average of 40517 reads per sample, resulting in 26 phyla, 49 classes, 92 orders, 111 families, and 204 genera. The unique numbers of OTUs in Ck, T1, T2, T3, T4, T5, and T6 were 840, 1055, 1036, 3807, 2234, 917, and 1236, respectively (Figure 3). Overall results showed that the unique number of OTUs was less in control and higher in combined biochar application with organic-inorganic fertilizers.

### Composition and community structure of the pomelo orchard microbiomes under different treatments

**FIGURE 4:**
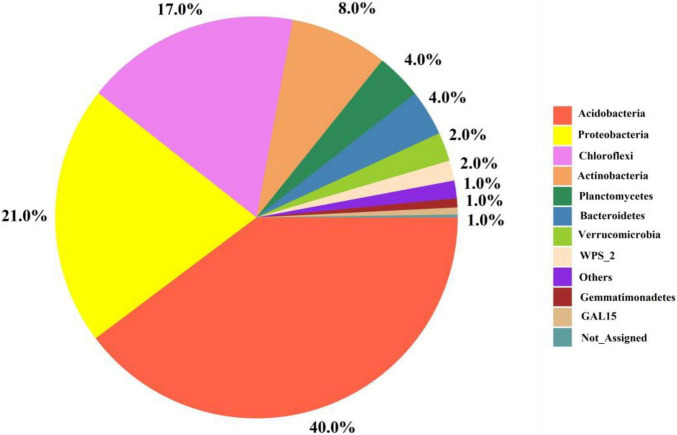
The interactive pie chart exploration of bacteria at phylum level among all soil samples.

**FIGURE 5:**
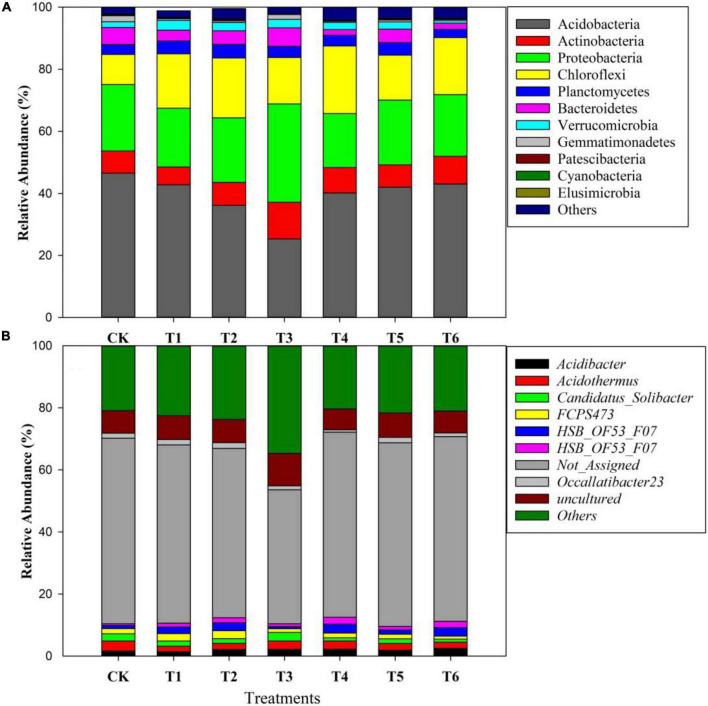
Changes in the relative abundance of soil bacteria at the phylum level **(A)** and genus level **(B)** among different treatments.

Figure 4 depicts the relative abundance of important bacteria at the phylum level in all soil samples. The most dominant bacterial phyla in the soil samples were Acidobacteria (40%), Proteobacteria (21%), Chloroflexi (17%), Planctomycetes (8%), Bacteroidetes (4%), Verrucomicrobia (2%), and Gemmatimonadetes (1%) among others (Figure 4). Furthermore, among the treatments, the relative abundance of bacteria at the phylum and genus levels are presented in Figures 5A,B. The results showed that Acidobacteria were abundant in all treatments (> 36%). The highest relative abundance of Acidobacteria was recorded in the control treatment (Ck) (46.5%) followed by T6 (43%) and T5 (42%). Increasing biochar rate up to 4 kg plant^–1^ (T3) decreased the relative abundance by 22% over the control treatment (Ck). The second most abundant bacteria were proteobacteria and were higher in organic-inorganic mixed fertilizer treatment (T3), ranging by 31.59% followed by Ck (21.35%) compared to other treatments. The lowest relative abundance of proteobacteria ranging by 17.38% was recorded in T4 (4 kg biochar + 1.7 kg organic-inorganic compound fertilizer) compared to all other treatments. Furthermore, Actinobacteria, Planctomycetes and Chloroflexi had maximum relative abundance, with range of 11.78, 11.78, and 4.38% in T4 and T3, respectively. Except for Acidobacteria and Proteobacteria, all other bacteria were higher in higher biochar rate than control.

The bacterial species at the genus level as affected by different biochar and organic-inorganic compound fertilizers are presented in Figure 5B. A total of 204 species were found among all treated and control soil samples. *Acidothermus* was the most abundant bacteria at genus level after others, not assigned and uncultured species, followed by *Acidibacter*, *Candidatus Solibacter* and *FCPS473*, the treatments showed that *Acidothermus* was highest in control treatment, followed by T4 and T5, while the lowest was recorded in biochar applied treatments (T2 and T3). In contrast, *Acidobacteria* species were increased in biochar applied soil compared to all other treatments. Furthermore, *Candidatus Solibacter* species were higher in control and T3, followed by T2 and T1, and the lowest was observed in T6.

### Alpha and beta diversity

Alpha diversity indices were generated for each sample to examine treatment diversity (Table 2). Alpha diversity Chao 1, ACE, Shannon and Simpson indexes were significantly affected by different treatments. Biochar treatments (T1 and T2) had a higher Chao 1 index than all other treatments, according to the findings. The lowest Chao 1 index was recorded in the control treatment (1082.5 ± 68.6c). Similarly, a higher ACE index (1206.7 ± 5.63) was observed in biochar applied treatment (T1), and the lowest was recorded in Ck.

**TABLE 2:** Alpha diversity index of soil microbial community in pomelo orchard treated with biochar.

Treatments	Chao 1 index	ACE index	Shannon index	Simpson index
Ck	1082.5 ± 68.6c	1073.7 ± 70.95c	5.21 ± 0.539c	0.977 ± 0.019c
T1	1217.8 ± 8.6a	1206.7 ± 5.63a	5.68 ± 0.120ab	0.990 ± 0.002a
T2	1203.4 ± 13.4ab	1187.4 ± 15.31b	5.74 ± 0.10a	0.993 ± 0.001a
T3	1086.8 ± 74.2c	1079.7 ± 69.95c	5.69 ± 0.091ab	0.993 ± 0.001a
T4	1111.6 ± 74.2b	1103.3 ± 62.41b	5.25 ± 0.284c	0.985 ± 0.005b
T5	1178.7 ± 11.7b	1173.1 ± 9.45b	5.58 ± 0.226b	0.986 ± 0.009b
T6	1161.5 ± 34.6b	1148.9 ± 28.95	5.39 ± 0.195bc	0.984 ± 0.009b

Different letters within the column indicates significant difference at P < 0.05. Ck (control), T1 (2 kg biochar plant^–1^), T2 (4 kg biochar plant^–1^), T3 (2 kg organic-inorganic mixed fertilizer plant^–1^), T4 (4 kg biochar + 1.7 kg organic-inorganic mixed fertilizer plant^–1^), T5 (4 kg biochar + 1.4 kg organic-inorganic mixed fertilizer plant^–1^), T6 (4 kg biochar + 1.1 kg organic-inorganic mixed fertilizer plant^–1^).

Furthermore, the Shannon index was higher (5.74 ± 0.120) in T2 and was lowest (5.21 ± 0.539) in Ck. Simpson index was not significantly affected by biochar, organic-inorganic fertilizers treatments. Overall results showed that biochar application increased the alpha diversity indexes.

**FIGURE 6:**
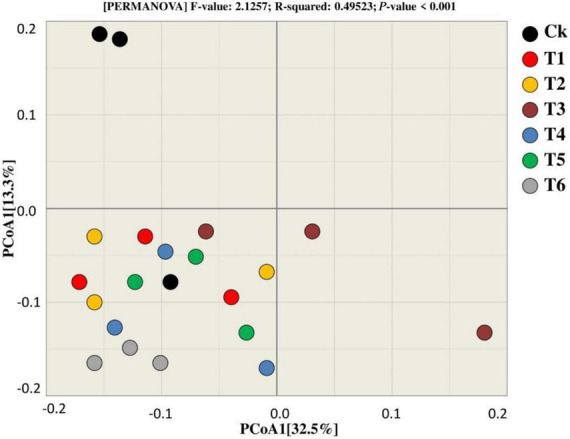
Beta diversity analysis was used for the bacterial community to estimate similarity and dissimilarity among treatments.

Principal coordinate analysis was utilized to compare and contrast the similarities and differences in bacterial beta diversity across all treatments (Figure 6). According to the findings, most of the samples from the respective treatments tend to clump together, showing that the treatments are similar because they cluster near each other, with the exception of Ck, which differs from the other treatments in terms of the bacterial community. Furthermore, PCoA1 accounted 32.5 percent of the variation among the treatments during both years, whereas PCoA2 explained 13.3% of the total variation among the treatments.

### Relationship between bacterial community composition, soil enzymes, and soil properties

**FIGURE 7:**
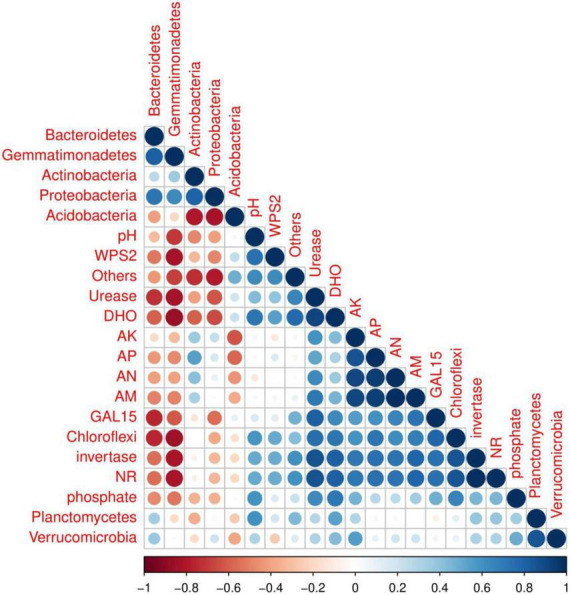
Correlation analysis of soil microbial abundance, soil characteristics, and soil enzyme activity among treatments.

Correlation among soil properties, enzymatic activities and soil bacterial abundance were determined by R software using the core plot package (Figure 7). The results showed that soil physiochemical properties affected by biochar application were significantly correlated to soil bacterial abundance and enzymatic activities. For example, strong negative relationships between soil pH and Gemmatimonadetes abundance and moderate negative correlated with Bacteroidetes, Actinobacteria and Proteobacteria abundance were recorded. However, among the treatments, a positive relation of soil pH with the relative abundance of Chloroflexi, planctomycetes, Verrucomicrobia, GAL15 and WPS_2 was recorded. Acidobacteria and proteobacteria were positively correlated with soil AK, AP, AN, and AM. Furthermore, soil enzymes activities, including DHO, NR, invertase, urease and phosphate were also recorded positively correlated with soil nutrients content and major soil bacterial abundance except Bacteroidetes and Gemmatimonadetes.

## Discussion

Pomelo orchard’s soil is severely degraded due to many factors such as soil acidification, low organic matter content and poor soil management (Van Dang et al., 2021). Fertilizers management, i.e., biochar, organic-inorganic fertilizer application, is considered a soil conservation measure that decreases soil acidification and degradation (Iqbal et al., 2022). Several studies documented the impact of biochar on plant growth, yield and soil health. However, the effect of biochar combined with organic-inorganic mixed fertilizers after 3 years on soil bacterial communities’ structure is still not clear. This research evaluates the impact of different biochar rates combined with various organic-inorganic fertilizers rates on pomelo orchard soil properties, enzymatic activities and bacterial community composition.

### Soil properties

Soil chemical properties such as pH, AK, AN, AP, and AM play an essential role in soil fertility and plant growth and development. In the present study, sole biochar and biochar combined with organic-inorganic fertilizer significantly improved soil pH compared to control and non-biochar applied treatments. The possible explanation for improving soil pH in biochar used soil might be due to biochar’s chemical properties; biochar itself has a high pH value, enhancing the pH of acidic soil. Similar results of improving soil pH by biochar application were reported by Imran (2021) and Ullah et al. (2021). In the current results soil AN, AP, and AM were improved in the combined treatment of biochar with organic-inorganic fertilizer (T4) as compared to all other treatments. The possible explanation of these increments might be due to the nutrient contents in both organic-inorganic fertilizers, while biochar holds these nutrients for a long time and is released slowly to plants for its requirements. In combination with other fertilizer, biochar amendment significantly increased soil enzymatic activities and soil microbial biomass (Lu et al., 2015; Ullah et al., 2021). Our results were supported by previous results of Arif et al. (2017); Naeem et al. (2018), and Singh et al. (2019), who reported that soil chemical properties were improved due to biochar application combined with organic-inorganic fertilizer as compared to their sole treatments. Overall, we observed that biochar improved the ability nutrients to stabilize in soil for a long time in pomelo orchard.

### Soil enzymatic activities

Soil enzymes such as phosphate, urease, DHO, NR and invertase activities are involved in several soil biochemical processes, for example, organic matter mineralization and other biogeochemical cycling of nutrients (Makoi and Ndakidemi, 2008; Oladele, 2019). In the present study, the results showed that sole biochar amendment significantly increased phosphate and DHO activities as compared to control. Similarly, urease, NR, and invertase activity improved considerably in combined biochar and organic-inorganic fertilizer compared to the control treatment. According to Harter et al. (2014), fluctuations in soil enzymatic activity impact soil processes such as nutrient cycling, organic matter breakdown, soil respiration, and N_2_O emissions. The higher soil enzymatic activity in biochar treatments is supported by Ullah et al. (2020) findings that a 40% enhancement in urease activity was observed in biochar combined with inorganic fertilizer treated soil. Previously it is reported that biochar application might increase (Ouyang et al., 2014; Ullah et al., 2020), decrease (Du et al., 2014) or have no effect on soil enzyme activities (Zhou et al., 2015) depending on the environment.

### Effect of biochar and organic-inorganic fertilizer on soil bacteria abundance

The diversity and richness of the microbial community are considered critical for soil integrity, functioning, and sustainability, yet they are frequently harmed by present farming techniques (Khan A. et al., 2022). In the current studies, different biochar rates combined with organic-inorganic fertilizers significantly influenced the soil bacteria and genus abundance (Figure 5). Compared to control (Ck), biochar amendment combined with organic-inorganic fertilizer significantly improved soil bacterial abundance. The possible reason for these increments due to increase in soil pH in biochar applied soils. Previously it has been well reported that soil physiochemical properties indirectly affect soil microbial abundance (Cole et al., 2019). For instance, soil pH is considered the most critical factor in affecting soil bacterial abundance (Liu et al., 2016; Yao et al., 2017; Zheng et al., 2019a,b; Sun et al., 2020). Thus, in the current experiment, biochar addition to acidic soil improved soil pH and resulting improvements in soil bacterial abundance. Similar results were reported by Ali et al. (2022) that biochar combined with N fertilizer enhanced soil bacterial abundance compared to control plots. Furthermore, another study documented that 40 t biochar ha^–1^ increased bacterial 16S rRNA gene copy numbers by 35–62% (Chen et al., 2015). Although Luo et al. (2017) and Gao et al. (2021) observed that biochar amendment in alkaline soil did not affect soil pH or bacterial abundance. Therefore, our results suggested that a reasonable rate of biochar combined with organic-inorganic fertilizer improved soil bacterial abundance in pomelo orchards.

### Effect of biochar and organic-inorganic fertilizer on community composition of soil bacteria

Biochar used in combination with other fertilizers, has been shown to have short- and long-term effects on bacterial community composition (Yao et al., 2017; Li et al., 2020). However, the impacts of biochar in combination with organic-inorganic fertilizer on pomelo orchard soil community composition are unclear. In the current study, the most abundant bacteria at the phylum level were Acidobacteria, Actinobacteria, Bacteroidetes, Chloroflexi, GAL15, Gemmatimonadetes, Others, Planctomycetes, Proteobacteria, Verrucomicrobia and WPS-2. Compared to other treatments, biochar combined with organic-inorganic fertilizers increased phyla Proteobacteria Actinobacteria, Bacteroidetes, and Verrucomicrobia, ranging from 31.51, 11.84, 5.88, and 2.71%. Proteobacteria accounts for the largest fraction of the soil community in terms of community composition and relative abundance, which is similar with the findings of Ji et al. (2016) and Yin et al. (2021). For detail Proteobacteria are eutrophic bacteria (Fierer et al., 2007). Biochar amendment has previously been shown to improve soil health (Ali et al., 2020a,b, 2021), resulting an increase in Proteobacteria population (Ali et al., 2022).

Acidobacteria and Gemmatimonadetes were higher in control plots ranging 46.53 and 1.9%, compared to all other plots, indicating that biochar and organic-inorganic fertilizer decreased Acidobacteria and Gemmatimonadetes. Acidobacteria and Gemmatimonadetes negatively correlate with soil pH (Ali et al., 2022). In this study, soil pH was enhanced in biochar-applied treatments compared to controls, which resulted in lower levels of Acidobacteria and Gemmatimonadetes. Similar reducing effects of biochar on Acidobacteria abundance was reported by Ali et al. (2022), while in high soil pH (8.50) was reported by Yin et al. (2021). Chloroflexi and GAL15 phyla were improved in T4 (4 kg biochar + 1.7 kg organic-inorganic mixed fertilizer/plant) treatment as compared to other treatments. The possible reason for these increments was that Chloroflexi and GAL15 were shown to be strongly positively linked with soil characteristics and enzymatic activities (Figure 7), which were improved by biochar and organic-inorganic fertilizers. As gram-positive bacteria, Actinobacteria play an important role in the breakdown of organic materials, including cellulose and chitin (Ali et al., 2019).

### Relationships between bacterial communities and soil quality traits

The addition of biochar and organic-inorganic fertilizer can influence soil physiochemical properties and biochemical properties, which can alter the bacterial community composition (Ali et al., 2022). In the present study, we observed that combined biochar and organic-inorganic fertilizer treatments significantly affected soil properties (Table 1) and soil enzymatic activities (Figures 1, 2). Figure 7 shows the positive relationship between soil nutrients content and enzymatic activities with the most abundant bacterial phyla. However, soil pH, urease, NR, phosphate, and DHO activities were negatively correlated with soil Gemmatimonadetes, Actinobacteria, and proteobacteria. A similar relationship of soil physiochemical properties with soil most abundant bacteria species was reported by Khan A. et al. (2022). Wu et al. (2020) reported that the structure and composition of the bacterial community were strongly positively correlated with soil quality attributes. Overall, the findings indicated that biochar at the rate of 2–4 kg plant^–1^ in combination with organic-inorganic fertilizer at the rate of 1.1–1.7 kg plant^–1^ might create a favorable environment for bacterial growth, hence increasing soil bacterial community structure and fertility.

## Conclusion

The results showed that organic-inorganic fertilizer combined with different biochar rates improved soil pH, increased nutrients contents, boosted enzymes activities and altered soil bacterial abundance and community structure. Compared to biochar and organic-inorganic fertilizer treatments, the bacterial Chao 1, ACE, Shannon, and Simpson indices were lowest in control. Furthermore, the lowest unique OTUs were also observed in the control plot and the highest was in combined treatments of biochar and organic-inorganic fertilizers. The correlation analysis showed that soil AK, AN, AP, and AM were positively correlated with soil most abundant bacterial phyla. Likewise, soil enzymatic activities (NR, DHO, invertase, phosphate) were also positively correlated with soil bacteria and soil properties. These findings were intended to serve as a guide and foundation for improving soil in pomelo orchards using a combination of biochar and organic-inorganic fertilizer with good application potential.

## Data availability statement

The original contributions presented in this study are included in the article/supplementary material, further inquiries can be directed to the corresponding author.

## Author contributions

YS and DL designed the study and wrote the manuscript. QZ, XG, and IA performed the data analysis and revised the manuscript. QZ, IA, XG, FL, and SL performed the data curation. All authors listed have made a substantial, direct, and intellectual contribution to the work, and approved it for publication.

## Footnotes

1
https://www.microbiomeanalyst.ca/
